# Dr. George Edward Fell

**Published:** 1918-09

**Authors:** 


**Dr. George Edward Fell,** Buffalo, 18. ., died in Chicago. . . . of paralysis, having been in bad health for several years. He was born in Chippewa, Ont., July 10, 1849. He was an engineer before becoming a physician. His life work may be summed up as follows:

Placed first crib of great Buffalo Breakwater June 8th, 1869. See Discussion, Trans. Am. Socy. Civil Engrs. Vol LII, p. 73, 1904.

Member 1st American Microscopical Congress, August, 1878, Indianapolis. Organizer of Am. Socy. Microscopists, at this Congress.

Elected first Treasurer and Custodian, Am. Socy. Microscopists, at the first meeting, Buffalo, N. Y., August, 1879. Held office 9 years.

Assisted in triangulation of floats for course and speed in location of draw-pier of International Bridge, as Asst. U. S. Engineer. 1879.

First Thesis on Histological Subject in Med. Dept. University of Buffalo, graduated with highest honors, Feby. 1882. Subject—"Histology of Aneurismal Clots."

Elected to the Chair of "Physiology and Microscopy," Med. Dept. Niagara University, held the office for about ten years. 1884.

First Discovered, after about 400 years of futile investigation of the ablest surgeons of previous history, the great value of mechanical respiration in saving human life in cases of asphyxia. He not only discovered but demonstrated by the most convincing cases the absolute advantage over the postural methods. The method has been in practical use since the date of the discovery in 1887. In many medical publications his work has been highly lauded by most noted members of the profession. He gave his discovery to the cause of humanity and has made nothing from his work.

First designer of a simple and practical steamboat pipe driver for making observation of the bottom of the lake at Buffalo and Cleveland Harbors, which did splendid service and repaid the U. S. Government many fold for its outlay. This was about 1883-1884. The apparatus was utilized for foundation purposes for the Buffalo Breakwater harbor construction.

Demonstrated in 1903 the mistake of building the seven million dollar plan of the present system of Water Works at Buffalo, N. Y.

Original observations of the Currents at the easterly end of Lake Erie and head of Niagara River demonstrated the truth of his statements and have all been upheld by later work of engineers employed by the Buffalo Chamber of Commerce, and later investigations which also proves the statements that Dr. Fell contended for in the face of great opposition. Opposing his enquiry were the State Commissioners of Health of the State of New York; the Health Commission of the City of Buffalo and nine-tenths or more of the physicians of Buffalo. See his publication, Journal Am. Med. Assn., Sept 3rd, 1910.

First used the face mask in mechanical respiration, thus doing away to a great extent with the necessity of tracheotomy. 1888-89.

The first to invent a simple, inexpensive, practical submarine life preserver using a face mask applied so as to be both air and watertight, this connected by a strong rubber or metal tube, to an upright, weighted air supply which extends well above the water and waves, and from which air is continuously supplied to the wearer of the mask. It has been proven successful and will accomplish much good. It may be used by both sexes and is simple in its application.

''Electrocution,'' which was first suggested by Dr. A. P. Southwick of Buffalo, N. Y., was made practical by the investigative work of Dr. Fell. He made the vivisection experiments which resulted in his conclusion, ''First—That death produced by a sufficiently powerful electrical current is the most rapid and humane produced by any agent at our command. ''Second—That resuscitation, after the passage of such a current through the body and functional centres of the brain, is impossible. ''Third—That the apparatus to be used should be arranged to permit the current to pass through the centres of function and intelligence in the brain.''

